# Spatial genetic structure and seed quality of a southernmost *Abies nephrolepis* population

**DOI:** 10.1038/s41598-023-45635-w

**Published:** 2023-10-27

**Authors:** Sunjeong Kim, Hye-Jin Lee, Yang-Gil Kim, Kyu-Suk Kang

**Affiliations:** Department of Agriculture, Forestry and Bioresources, College of Agriculture and Life Sciences, Seoul National University, Seoul, 08826 Republic of Korea

**Keywords:** Population genetics, Natural variation in plants, Plant genetics

## Abstract

*Abies nephrolepis* (Trautv. ex Maxim.) Maxim. has its southernmost populations in South Korea and they are expected to decline under climate change. To establish a strategic conservation plan, this study aimed to investigate the spatial genetic structure and seed characteristics of *A. nephrolepis*. We used nine microsatellite markers on 165 individuals of *A. nephrolepis* and sampled seeds in a southernmost population at Mt. Hambaeksan, South Korea. We observed a high level of heterozygosity, and a simulation study found that sampling 20 individuals was enough to secure sufficient genetic diversity on average. Spatial autocorrelation analysis revealed that individuals had a positive genetic relationship until 30 m. Bayesian clustering models, STRUCTURE and GENELAND, failed to achieve a consensus in the optimal number of population (K), estimating K = 1 and K = 2, respectively. Principal coordinate analysis supported the absence of genetic substructure within the study population. There was a large variance in seed production among mother trees. On average, seeds of *A. nephrolepis* from Mt. Hambaeksan had a purity of 70.4% and a germination percentage of 32.2%. We found that seed weight was the most effective indicator of seed quality. Mother trees at higher altitudes had poorer purity which is threatening to *A. nephrolepis* considering the upslope retreat of subalpine species under climate change. Our results provide insights into the interactions among spatial processes, genetic structure, and seed quality within a population of *A. nephrolepis*.

## Introduction

In forest trees, 90% or more of the total genetic diversity of a species is often found within individual populations^1^. Understanding the spatial genetic structure (SGS) in a population is important because spatial structure influences genetic parameters including mating system, selection, and genetic drift^2^. Investigating and comparing the SGS through generations offers important clues about historical and demographic forces that have affected the population^3,4^. Spatial autocorrelation analysis offers information about the SGS by comparing the structure of a population to a random arrangement and has been applied to various studies^5^. Bayesian clustering models describe multi-locus genotypes as clines or clusters to infer the SGS or recently created genetic barrier^6,7^. Comparing multiple Bayesian clustering models, including both spatial and non-spatial, is informative with additional independent inference methods, such as principal component analysis (PCA) or principal coordinate analysis (PCoA)^8^. For the investigation of genetic variation, microsatellite markers are commonly used on trees. Microsatellites, also known as simple sequence repeats (SSRs), are highly reproducible, polymorphic, codominant markers^9^. Microsatellite markers are useful to estimate the relationship or relatedness among individuals of unknown ancestry^10^ and have an advantage of cost-effectiveness^11^.

Integration of genetics into conservation is getting more important as genetic diversity critically contributes to the survival of a species^12,13^. Ex-situ conservation has a critical role in providing materials for restoration and preventing extinction, so it gets highly important when additional threats can lead the species to an extinction vortex^14^. The materials for ex-situ conservation need to be collected from distanced trees to avoid inbreeding and encompass the genetic variation that target populations have^15^. Typically, the goal of gene conservation is to capture at least one copy of 95% of the common alleles that occur in target populations at frequencies over 0.05^15^, and it is impractical to set the goal to include whole alleles because it requires a much larger sampling size^1^. The sampling size to reach this goal differs by species because of the distinct characteristics of each species, including the mating and pollination systems^1^. Therefore, to establish an appropriate sampling strategy for the target species, we need to understand the pattern and extent of the genetic variation among and within its populations.

For the restoration and conservation of tree species, seeds are the primary source of propagation because most tree species rarely propagate asexually in nature^16,17^. Seed characteristics need to be understood to efficiently collect the germplasm for conservation. Seed quality is a major factor that affects the seed sampling strategy^17^. The important indicators of seed quality include purity, germination percentage, and viability. While germination percentage is more easily tested, some researchers^18^ argue that viability is more important. They noted that germination percentage can provide imprecise information because we lack knowledge on dormancy and germination. Genetic diversity or genetic structure of the population is another important factor for the establishment of a good seed sampling strategy. An increase in homozygosity leads to embryo death, more empty seeds, and lower viability in various species, including *Abies*^19^. Therefore, genetic diversity of the population is at the core of sustainable reproduction and regeneration.

*Abies nephrolepis* (Trautv. ex Maxim.) Maxim. is a wind-pollinating conifer belonging to the section *Balsamea* of *Abies*^20^. It is naturally distributed in the subalpine or boreal forests of eastern Russia, northeast China, and the Korean peninsula^21,21^. It was assessed as Least Concern (LC) in 2010 (published in 2013) according to IUCN Redlist^22^. The main threat to the species is known to be logging^21^, but recently, the populations in South Korea have been affected by climate change. The Korea Forest Service noted that *A. nephrolepis* showed a decline rate of 31% in 2019–2020, which was estimated to be caused by higher temperature and droughts^23^ It is expected that the potential habitats of *A. nephrolepis* on the Korean peninsula will decrease to 36.4% by 2070–2099, and most regions in South Korea were expected to be inhabitable^24^. Populations of *A. nephrolepis* in South Korea had a low level of genetic differentiation and only about 3–8% of total genetic variation resulted from the variation among populations^25–27^. These studies suggest that we could make the conservation more efficient by focusing on several populations.

Our goal was to produce insights that lead to the establishment of an effective conservation plan in a southernmost population of *A. nephrolepis*. For this, this study had two main objectives: (1) to understand the within-population genetic variation in *A. nephrolepis* by investigating the genetic diversity and SGS, and (2) to identify the characteristics of seeds in a population. The study was conducted at Mt. Hambaeksan, South Korea, which was the southernmost population having dominant mitochondrial DNA haplotypes of *A. nephrolepis*^28^. We will discuss the relationships among genetic variation, seed quality, and spatial process.

## Results

### Genetic diversity and sampling simulation

Genetic diversity was quite high in the population of *A. nephrolepis* at Mt. Hambaeksan (Table 1). Among the nine microsatellite markers, two markers, AK171 and AK252, were estimated to have the null alleles (estimated frequency over 0.05). However, null alleles were uncommon with frequency under 0.2, so we considered it acceptable for further analysis^29^. All amplified loci were polymorphic having over three effective alleles. All individuals were distinguished by their genotypes using any combination of three markers. A Mantel test showed that there was no significant isolation by distance (IBD) (R^2^ = 0.0002^NS^, NS: not significant). Genetic diversity indices were also calculated on two different diameter classes (Table 2). The mean number of effective alleles and the mean expected heterozygosity of each class were similar. The group of bigger trees (BT) showed a relatively lower mean number of alleles, observed heterozygosity, and fixation index, but within the error range.

**Table 1:** Genetic diversity indices estimated in the population of *A. nephrolepis* at Mt. Hambaeksan, South Korea over nine microsatellite loci.

Locus	Size range (bp)	Null allele frequency	*A*	*A*_*E*_	*H*_*O*_	*H*_*E*_	*F*
AK87	280–324	− 0.018	21	10.789	0.939	0.907	− 0.035
AK171	220–252	0.175	15	3.613	0.627	0.723	0.133
AK173	176–216	0.016	18	7.511	0.836	0.867	0.035
AK176	328–354	− 0.020	7	4.583	0.812	0.782	− 0.039
AK246	166–176	0.022	6	3.885	0.709	0.743	0.045
AK247	184–206	0.037	12	9.159	0.824	0.891	0.075
AK252	305–237	0.138	11	5.320	0.787	0.812	0.031
As13	244–272	− 0.028	12	5.978	0.879	0.833	− 0.055
As20	192–236	− 0.028	20	5.617	0.867	0.822	− 0.054
Average			13.556	6.273	0.809	0.820	0.015
SE			1.788	0.811	0.031	0.021	0.022

*A*, number of alleles; *A*_*E*_, number of effective alleles; *H*_*O*_, observed heterozygosity;* H*_*E*_, expected heterozygosity; *F*, fixation index; SE, standard error.

**Table 2:** Genetic diversity indices of different diameter class of *A. nephrolepis* at Mt. Hambaeksan, South Korea.

		*A*	*A*_*E*_	*H*_*O*_	*H*_*E*_	*F*
All individuals	Mean	13.556	6.273	0.809	0.820	0.015
	SE	1.788	0.811	0.031	0.021	0.022
BT	Mean	12.333	6.204	0.789	0.814	0.036
	SE	1.624	0.813	0.046	0.026	0.034
ST	Mean	13.000	6.202	0.828	0.819	− 0.011
	SE	1.700	0.784	0.024	0.020	0.023

*A*, number of alleles; *A*_*E*_, number of effective alleles; *H*_*O*_, observed heterozygosity;* H*_*E*_, expected heterozygosity; BT, bigger trees; ST, smaller trees; SE, standard error.

According to the sampling simulation study, sampling 20 individuals ensured capturing over 95% of the total common alleles on average (Supplementary Fig. S1). When we checked the success rate of capturing sufficient common alleles (over 95%) at every locus, sampling individuals only showed 31.1% of the success rate (Supplementary Fig. S1). At least 25 individuals were needed to make over 50% of the samplings to be successful, and 40 individuals were required to exceed 90% (or 95%).

### Spatial genetic structure

**Figure 1:**
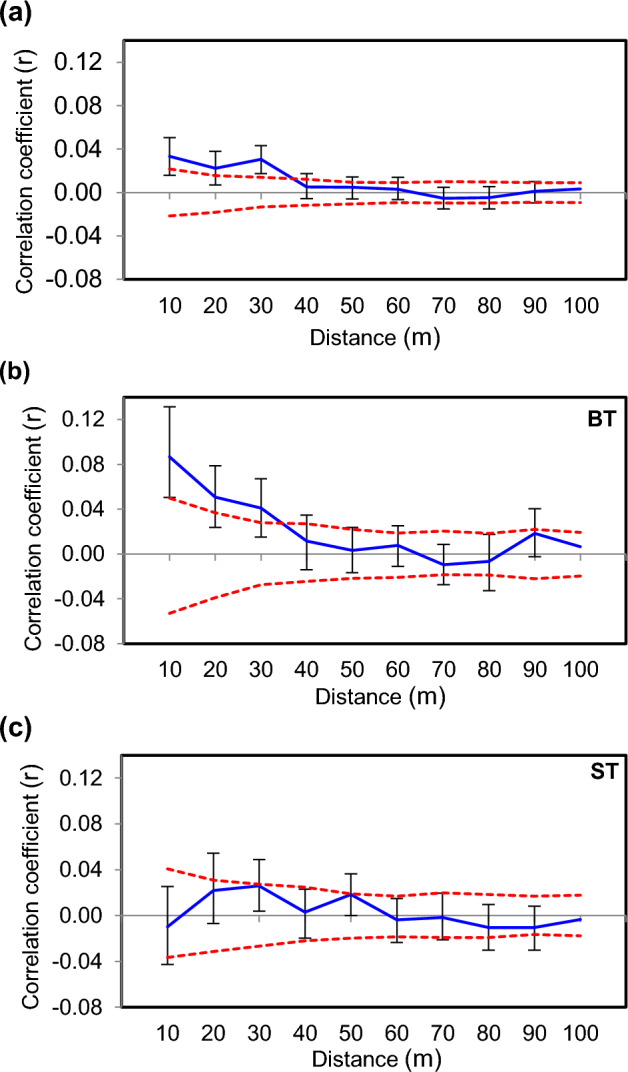
Correlogram of the population of *A. nephrolepis* at Mt. Hambaeksan in 10 m distance intervals. The blue line represents values of correlation coefficient (r) against the geographical distance between individuals with black error bars of 95% confidence interval. The red dotted line indicates the upper and lower limits of the null hypothesis with a 95% confidence interval. (**a**) Correlogram of all individuals, (**b**) correlogram of the bigger trees (BT), and (**c**) correlogram of the smaller tree (ST).

We found a weak SGS within the study population of *A. nephrolepis* at Mt. Hambaeksan. Significantly positive SGS was found up to 30 m (Fig. 1a). Individuals were in genetic randomness from the distance interval of 30–40 m, and until 400 m which was approximately the farthest distance between the trees (Supplementary Fig. S2). When we investigated SGS by diameter class, the group of bigger trees (BT) showed a similar result to that of all individuals (Fig. 1b). They had significantly related individuals until 30 m and distributed in genetic randomness from the distance interval of 30–40 m. In contrast, the group of smaller trees (ST) did not show significant SGS in all distance classes (Fig. 1c).

The *Sp* value obtained from all individuals was 0.0036 (*F*_*1*_ = 0.0103***, *b*_*F*_ = − 0.0036***, ***p < 0.001). When the values were calculated by the diameter group, BT had *Sp* = 0.0081 (*F*_*1*_ = 0.0270***, *b*_*F*_ = − 0.0079***), which was higher than the value obtained from all individuals. However, ST had a lower value of *Sp* = 0.0021 (*F*_*1*_ = 0.0030^NS^, *b*_*F*_ = − 0.0021^NS^), and it was not statistically significant. It indicated that the bigger trees had a greater level of SGS. Overall, SGS found in all individuals could have resulted from the SGS in the bigger trees.

**Figure 2:**
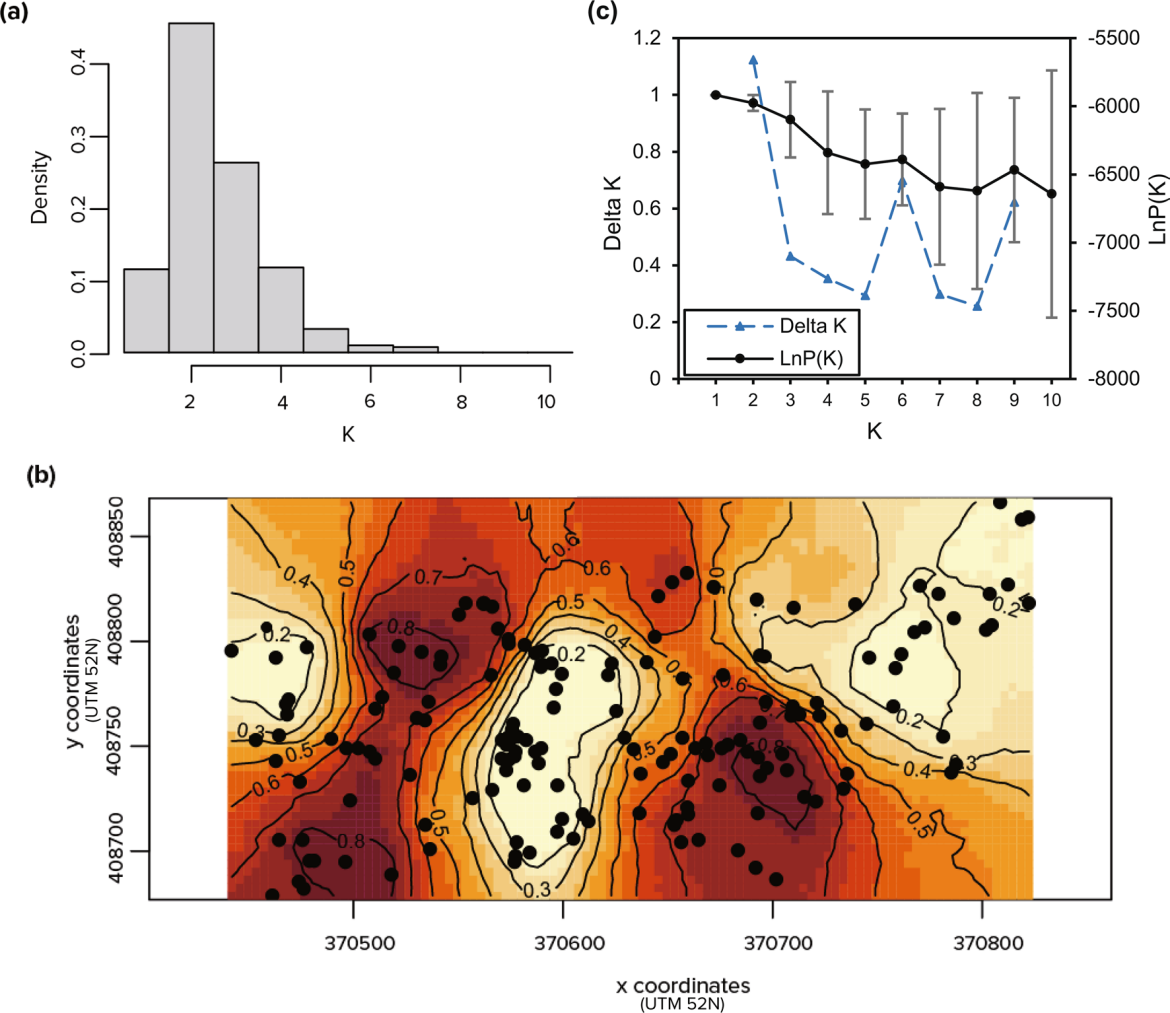
Results of Bayesian clustering models of the population of *A. nephrolepis* at Mt. Hambaeksan, South Korea. (**a**) The optimal number of clusters estimated from GENELAND, (**b**) map of posterior probability estimated from GENELAND, and (**c**) the optimal number of clusters estimated from STRUCTURE. X and y coordinates are the geographical coordinates of the individuals in Universal Transverse Mercator (UTM52N).

The optimal number of clusters (K) inferred by two Bayesian clustering models, GENELAND and STRUCTURE, was inconsistent. In 20 independent runs in GENELAND, all runs produced consistent results, K = 2 (Fig. 2a, b). However, the two clusters had an *F*_*ST*_ of 0.009 (p = 0.001), which was a very low level of differentiation. Principal coordinate analysis (PCoA) failed to demonstrate a clear clustering that coincides with the GENELAND result (Supplementary Fig. S3). The best K inferred by STRUCTURE was different depending on the methods. Pritchard’s method presented K = 1 as the best K, but Evanno’s ΔK method presented K = 2 (Fig. 2c). PCoA did not demonstrate a clear clustering that coincides with the result of STRUCTURE of K = 2 (Supplementary Fig. S3). In this study, K = 1 by Pritchard’s method was chosen as the optimal number of clusters estimated by STRUCTURE.

### Cone and seed characteristics

The 31 mother trees included all common alleles and 76.61% of total alleles. On average, the mother trees had a diameter at breast height (DBH) of 14.55 cm (± 3.86 cm) and the smallest tree had a DBH of 5.2 cm. The mother trees produced 55.77 (± 67.36) cones on average, and there was a huge variance among trees. The least productive tree only produced five cones, while the most productive one produced about 270 cones. Overall, the top 20% of the most productive mother trees produced 61% of the total cones, and 81% of the cones were produced by the top 42% of the mother trees.

The cone and seed characteristics of the sampled mother trees (Table 3) were similar to a previous study that analyzed 5 different populations in South Korea^30^. However, the purity was approximately 10% lower than the forest seed standard quality investigated by the Forestry Research Institute of South Korea^31^, which is now known as the National Institute of Forest Science.

**Table 3:** Cone and seed characteristics resulted from the cone analysis in the population of *A. nephrolepis at* Mt. Hambaeksan, South Korea.

	CL (mm)	CW (mm)	DW (g)	W100 (g)	SP	DS	Purity (%)	P-DS (%)	P-DM (%)
Mean (± sd) of this study	55.35 (± 6.90)	23.4 (± 1.66)	6.578 (± 1.888)	0.958 (± 0.184)	278.4 (± 43.9)	259.3 (± 44.7)	70.2 (± 6.6)	93 (± 4.7)	10.4 (± 11.4)
Range (min–max) of this study	36.95–71.83	19.68–26.23	3.433–11.392	0.504–1.433	174–378	158–365	48.7–81.2	73.1–100.0	1.1–62.9
Average range of other pops^30^	55.60–64.50	20.80–23.40	–	0.700–1.100	–	–	–	–	–
Standard quality^31^	–	–	–	0.930	–	–	82	–	–

CL, cone length; CW, cone width; DW, dry weight of cone; W100, 100-seed weight; SP, seed potential; DS, number of developed seeds; P-DS, percent developed seeds; P-DM, percent damaged seeds; sd, standard deviation; min, minimum; max, maximum; pops, populations.

Detailed definitions of the terms are given in Supplementary Table S2.

Benefiting from the long pre-chilling period, seeds germinated early (Supplementary Fig. S4). A seed was first germinated only a single day after the germination test began. The mean germination time was 7.4 days and the germination energy on day 6 was 15.4%. In the end, the germination percentage was 32.2% and the viability was 34.2%. The survivorship of 480 seedlings after 2 months was 77.5% and the survivorship after 6 months dropped to 64.2%.

### Correlation among seed characteristics

**Figure 3:**
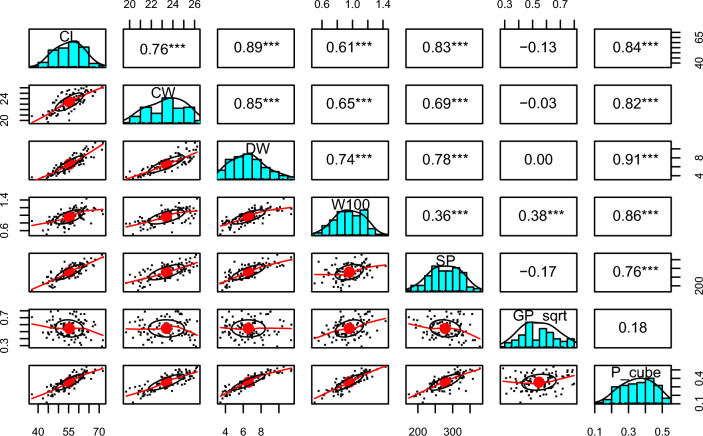
Results of correlation analysis on cone characteristics in the population of *A*. *nephrolepis* at Mt. Hambaeksan, South Korea. CL, CW, DW, W100, SP, GP_sqrt, P_cube each represent cone length, cone width, dry weight 100 seed weight, seed potential, germination percentage after square root transformation, and purity after cube transformation. ***p < 0.001.

There were significant relationships between cone characteristics (Fig. 3). The cone length, cone width, dry weight of a cone, 100-seed weight, and seed potential had positive relationships with each other. All of them also had a positive relationship with purity. However, when it comes to the germination percentage, only 100-seed weight had a significant relationship.

There were also several significant relationships between the mother tree characteristics. The DBH and cone abundance had a positive relationship (r = 0.6053***), and the geographic position was also related to seed quality and quantity (Supplementary Fig. S5). Mother trees at higher altitudes produced more cones (r = 0.4037*, *p < 0.05), but they had lower purity (r = − 0.3905*). We observed a weak tendency between genetic distance and seed quality (Supplementary Fig. S6), however, correlation tests failed to discover a significant relationship. Neither genetic distance (r = 0.3145^NS^, p = 0.08) nor the number of trees within the distance of spatial autocorrelation (30 m) (r = − 0.1900^NS^, p = 0.31) was not significantly related to the seed quality.

## Discussion

### Fine-scale spatial genetic structure

In the result of STRUCTURE, the optimal number of clusters estimated by Pirtchard’s method and Evanno’s ΔK method were different from each other (K = 1 and K = 2, respectively). Although Evanno’s ΔK method was developed to improve the detection of the optimal number, it cannot calculate the value at K = 1, therefore Evanno’s ΔK method selects K = 2 more frequently^32^. Some researchers also evaluated that Evanno’s ΔK method only brings little improvement compared to Pritchard’s method^33^. The two Bayesian clustering models, GENELAND and STRUCTURE, also failed to achieve consensus in estimating the optimal number of K within the population. There were several studies reviewing multiple Bayesian clustering models together^34,35^, and they reported that a low level of genetic differentiation can increase the error rate in the Bayesian clustering model. The two clusters found with GENELAND in *A. nephrolepis* at Hambaeksan had a very low level of differentiation, and this could have increased the error rate. Also, although the correlated frequency model is better for the populations with low differentiation, researchers noted the problem of poorer accuracy^35,36^, and previous studies reported overestimation of K under the correlated frequency model in GENELAND^37,38^. Also, the results of PCoA supported the absence of clustering within the population, and there was no apparent abiotic barrier that can explain the result of GENELAND. For these reasons, we concluded that the correlated frequency model of GENELAND overestimated K, and the genetic clusters had not yet developed within the population.

SGS in the population of *A. nephrolepis* at Mt. Hambaeksan was not significant and the estimated *Sp* value (0.0036) was relatively weak compared to the average of outcrossing species (0.0126) or wind-dispersed species (0.0064)^39^. However, it was akin to *Sp* values of other species in *Abies*^40,41,42^. While there was no significant SGS in ST, BT had a stronger SGS. Previous studies found that younger generations of outcrossing species can have a weaker SGS^43–45^. According to these studies, an increase in genetic structure towards older life-history stages can result from local selection, historical events, and demographic factors. In this study, BT showed a higher level of inbreeding coefficient (*F* = 0.036) compared to ST (*F* = − 0.011). Considering the existence of a transmitting station right near the study area, populations of *A. nephrolepis* in the study area could have experienced a bottleneck event caused by the anthropogenic disturbance, then slowly recovered the genetic diversity by active gene flow in younger generations. However, we could not observe a clear decrease in the number of alleles in BT (Table 2) which is the major evidence of bottleneck events^45,46^. Therefore, it was hard to conclude what caused the difference in SGS between the diameter class of *A. nephrolepis* at Mt. Hambaeksan. Moreover, although DBH is commonly used as an indirect indicator of age, it is hard to compare the diameter class directly to the age structure. Among the sampled individuals in this study, we extracted cores from the two trees that belonged to different diameter classes, but they were estimated to have similar ages (Supplementary Table S3). To study the change in SGS through generations, we should sample individuals in various life-history stages, including seedlings, saplings, and juveniles, or investigate the age structure more directly using cores.

### Seed quality

The seeds and cones of *A. nephrolepis* at Mt. Hambaeksan mostly had similar characteristics to other populations, but the purity was lower than the standard quality. Considering that the standard quality was published in 1994, changes in climate conditions could have resulted in poorer purity. According to the data from the nearest weather station^47^, the sampling year had a higher temperature and less precipitation compared to the normal year (past 30 years): the average annual temperature was about 0.5℃ higher, and annual precipitation was about 100 mm less. There were several studies on the relationship between climate change and seed quality. They found that the increased temperature and moisture stress can reduce the seed yield and quality, causing shortened duration of stigma receptivity^48^. Temperature irregularity affects the flowering timing, which causes asynchronous flowering within a population^49^. Therefore, the increased temperature might have affected pollen production or pollination of *A. nephrolepis*. Especially, the genus *Abies* lacks pollination drop and uses rainwater as an alternative^19^. Therefore, decreased precipitation could have decreased pollination success.

The correlation analysis between seed characteristics found that more cones existed in trees with bigger DBH. This result corresponds to the previous research that noted, for some species in Pinaceae, older trees produced more cones while younger trees produced more pollen^50^. The amount of pollen of *A. nephrolepis* was not measured in this study, therefore, further studies might offer interesting information about the relationship between age and strobili production. There was a positive correlation between seed weight and seed quality. With this result, prioritizing heavier seeds in propagation seems efficient. For sampling, direct evaluation of seed weight is impossible because it needs post-collection treatment, such as drying or cleaning. Cone weight can be used as a good alternative because it had the strongest correlation with the seed weight and also had a positive relationship with the purity.

Among the geographic factors, altitude showed a relationship with purity and cone abundance. The decrease in purity along the altitude could have been caused by the decrease in pollen pool. As the study plot was located right below a transmitting station on the summit, the individuals at the high altitude were on margin, therefore had a decreased pollen pool, which leads to poor reproductive success. This leaves important implications in seed production and regeneration of *A. nephrolepis* under climate change. Several studies expected that the lower limit of subalpine or alpine conifers will retreat upward due to climate change and have a decrease in its central area. The lowest limit of regeneration of *Abies georgei* Orr had retreated upslope 31 m per decade^51^, and *A. nephrolepis* was expected to lose its lowlands habitats^52^. Considering a decrease in purity at higher altitudes of Mt.Hambaeksan, loss and upward-retreat of habitable areas will result in poor regeneration of *A. nephrolepis* in South Korea. Therefore, the need for ex-situ conservation gets more important to adapt to climate change.

The correlation analysis failed to find a significant relationship between genetic relatedness and seed quality. Because the genus *Abies* has developed a post-zygotic barrier, it was expected that mother trees having more genetically close pollen trees would have poorer seed quality than the opposite. The insignificance could be because of the sufficient gene exchange among the mother trees. *A. nephrolepis* was a dominant species in the study plot and occupied the upper canopy. As *A. nephrolepis* is a predominantly outcrossing wind-dispersed conifer, occupying the upper canopy allows active pollen dispersal.

### Strategy for ex-situ conservation

With this study, we could establish several sampling and management strategies for *A. nephrolepis* at Mt. Hambaeksan. For the ex-situ conservation of *A. nephrolepis* at Mt. Hambaeksan, materials need to be collected from distant individuals that are at least 30 m away to avoid genetic relatedness. To encompass sufficient genetic diversity, at least 20 individuals have to be sampled within the study population. However, to secure the common alleles at every locus, much more individuals need to be sampled. Keeping sampling individuals distant, over 30 m away, will help reduce the optimal number of sampling. In this study, the 31 mother trees, which were 35 m apart from each other on average, included all common alleles.

We could also establish some strategies for seed collection. Although trees with bigger DBH had more cones in a tree, it did not ensure seed quality or genetic diversity. Therefore, cones must be collected from various distant trees, regardless of size. However, the geographical position of the mother trees should be considered. It is better to avoid mother trees on the margin because they could have less gene exchange and lower purity. Within a tree, collecting heavier cones would be effective, as heavier cones have a higher probability of good quality. After the seed cleaning, heavier seeds should be chosen, as they are more likely to germinate.

*Ex-situ* conservation is more emphasized and important for the species under threat, however, for the effective conservation of *A. nephroelpis*, *in-situ* conservation should be carried out together. A previous study on the conservation of *Taxus cuspidata* Sieb. et Zuuc., a species with a similar genetic and geographical distribution pattern to *A. nephrolepis*, pointed that *ex-situ* gene conservation might reduce overall genetic diversity and population size^53^.

## Conclusions

This study aimed to understand the genetic variation and seed characteristics in a southernmost population of *A. nephrolepis*. The flowering individuals had a positive genetic relationship until 30 m, so we concluded 30 m is the optimal sampling distance of *A. nephrolepis* at Mt. Hambaeksan. The overall extent of the SGS was weak, but it was stronger among individuals in a bigger diameter class. There was no cluster found within the study population, therefore we considered sampling 20 individuals over the site would be enough to secure the genetic diversity of the population. The cones and seeds had similar characteristics to other populations in South Korea. Correlation analysis revealed that the DBH was positively related to the cone abundance and the seed weight was the most effective indicator of the seed quality. The purity was relatively low, and it got lower as the altitude got higher. Mother trees that were genetically closer to other individuals seemed to have poorer seed quality, but it was insignificant.

This study investigated SGS and seed quality together in a population of *A. nephrolepis*. This study contributes to the establishment of an effective ex-situ conservation of *A. nephrolepis* in a southernmost population in South Korea. Further studies including demographic and historical factors, mating systems and pollen dispersal need to be continued for a better understanding of the species. The impact of climate change on the regeneration of *A. nephrolepis* needs to be studied, as we inferred that higher temperature and upward-shifting reduction of habitats could disturb the healthy seed production.

## Materials and methods

### Sampling

**Figure 4:**
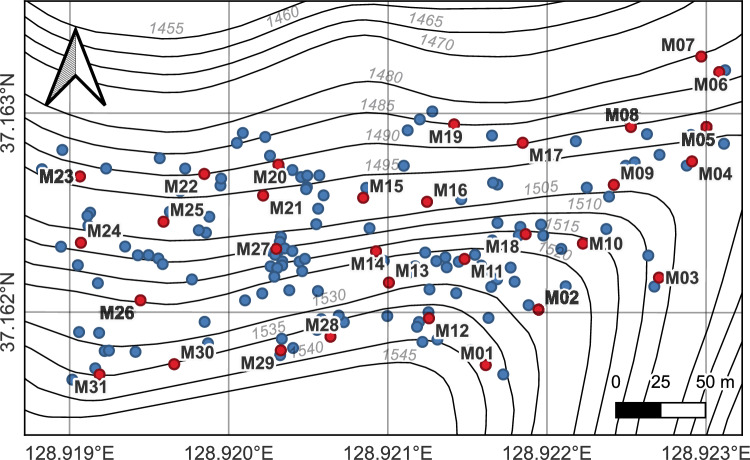
Geographic positions of the sampled *A. nephrolepis* at Mt. Hambaeksan. Dots indicate the location of sampled trees. Mother trees were marked by red dots with bold letters. Gray ray italic letters indicate altitude (m). Map of contour line was created by National Geographic Information Institute^56^.

Sampling was carried out in a natural population of *A. nephrolepis* at Mt. Hambaeksan, South Korea. The study area was about 5 ha, located on the northern aspect near the mountaintop (latitude: 37.161° N–37.164° N, longitude: 128.918° E–128.924 ˚E, altitude: 1482–1545 m). Within the plot, all mature flowering individuals were sampled. *A. nephrolepis* forms strobili and pollinates in April to May, and its seeds mature in September to October^54^. We defined a mature flowering tree as a tree with cones (strobili) at the time of sampling. We collected fresh needles from 165 individuals (Fig. 4) in late September 2021, and measured their height and diameter at breast height (DBH). Cones were sampled from 31 mother trees within the same plot in late September 2021 (Fig. 4). The average neighboring distance of the mother trees was 35 m. We collected at least three cones from each mother tree and a total of 141 cones were collected. Geographic positions (latitude, longitude, and altitude) were recorded in coordinates within a 3–4 m error range using Garmin GPSMAP 64 s (Garmin, Olathe, KS, US) and drawn on the map using QGIS 3.28^55^. All methods were carried out in accordance with the IUCN policy statement on research involving species at risk of extinction. Sampling was carried out with the permission of the Korea National Park Service. Our research team and Dr. Suh G.U. from the Korea National Arboretum undertook the formal identification of the plant material used in our study. The voucher specimen of the studied material has been deposited in the herbarium of the Korea National Arboretum (the deposition number: KNKA201106145001, KNKA201106145006, KNKA201106145007).

### DNA extraction and PCR amplification

DNA was extracted from the fresh needles using QIAGEN DNeasy Plant mini kit (QIAGEN, Hilden, Germany) and GeneAll Exgene Plant SV mini kit (GeneAll, Seoul, Korea). We followed the protocol of each kit with slight modifications. The modifications included increasing the time of 65 ℃ incubation and ice incubation to 30 min. The concentration and quality of the genomic DNA were measured with NanoVue Plus (Biochrom, Holliston, MA, USA).

For genotyping, nine microsatellite markers, AK87, AK171, AK173, AK176, AK246, AK247, AK252^57^, As13, and As20^58^ (Supplementary Table S4), were selected based on their polymorphism and amplification pattern. PCR amplifications were performed with a total volume of 12 μl containing 20 ng DNA, 1 X A-Star Taq Reaction Buffer, 2.5 mM MgCl_2_, 0.2 mM dNTP Mix, 0.5 U A-Star Taq DNA polymerase (BioFact, Daejeon, Korea), 0.04 μM HEX/FAM M13(-19) primer, and 0.2 μM of each reverse and forward primer. PCR programs were performed as described in each reference.

Fragment analysis was performed with PCR products by the National Instrumentation Center for Environmental Management (NICEM) in Seoul National University, with GeneScan 500 ROX size standard and ABI 3730 XL DNA Analyzer (Applied Biosystems, Waltham, MA, USA). Genotypes were scored using Microsatellite 1.4.7 plugin in Geneious Prime 2022.1.1^59^. Large allele dropout, stutter allele, and null allele were checked and adjusted using MICRO-CHECKER^60^ with the Oosterhout algorithm, 1000 permutations.

### Genetic diversity and sampling simulation

Genetic diversity indices were calculated using GenAlEx 6.503^61^. The number of alleles (*A*), number of effective alleles (*A*_*E*_), observed heterozygosity (*H*_*O*_), expected heterozygosity (*H*_*E*_), and fixation index (*F*) were calculated. To identify the degree of isolation by distance (IBD), we performed a Mantel test with 999 permutations between genetic distance and geographic distance of individuals.

A sampling simulation study was performed to find the minimal sampling size that can reach the genetic diversity. Here, the simulation goal was to secure over 95% of common alleles, which appear at over 0.05 frequency in the populations. If the simulated sampling reached the goal, we defined it as “success”. The sampling simulation study was carried out in Python 3.10.6^62^. Individuals were randomly sampled with a sampling size of 5–50 and checked whether they confirmed the goal (“success”). The random sampling was permuted 1000 times at each sampling size, and we counted the number of successes during 1000 permutations.

### Spatial genetic structure

SGS was investigated by three methods: spatial autocorrelation analysis, *Sp* statistic and Bayesian clustering model. This study only sampled adult trees, but we checked whether SGS differs by diameter class within the same life-history stage. To group the individuals into two diameter classes, the median (15.3 cm) of the DBH was used as a criterion. Individuals bigger than the median were grouped into the bigger trees (BT; 84 individuals with an average of 19.2 cm), and the others were grouped into the smaller trees (ST; 81 individuals with an average of 12.4 cm).

For spatial autocorrelation analysis, the correlation coefficient (r) of Smouse and Peakall^63^ was used. The correlation coefficient was calculated using GenAlEx 6.503^61^. The analysis was performed on even distance classes (10 m, 20 m, 40 m). Correlograms were plotted at the end point of the distance interval. Statistical significance of r values and correlograms were determined using 999 permutations and 1000 bootstraps, respectively.

*Sp* statistic, developed by Vekemans and Hardy^39^, enables the comparison of the extent of SGS among different populations or species. *Sp* statistic estimates SGS with regression slope of kinship coefficient (*F*_*ij*_) against spatial distance: ${S}_{p} = -{b}_{F} / (1-{F}_{1})$, where *b*_*F*_ is the regression slope of kinship coefficient on the log of distance and *F*_*1*_ is the mean of *F*_*ij*_ between individuals belonging to a first distance interval. SPAGeDi 1.5^64^ was used to calculate the *Sp* statistic. *F*_*ij*_ of Loiselle et al.^65^ was used, with an even distance class (20 m). Statistical significance of *b*_*F*_ and *F*_*1*_ was determined with 10,000 permutations.

To identify the genetic clusters within the study population, two different Bayesian clustering methods were used: GENELAND^66^ and STRUCTURE^67^. GENELAND 4.9.2 was performed in R 4.2.0 environment^68^. Twenty independent Markov chain Monte Carlo (MCMC) runs were performed with 500,000 iterations and 500 thinning intervals. Spatial model and null allele model were assumed under the possibility of having 1–10 populations (K). Correlated frequency model was selected, as it is more appropriate to detect subtle structures with a low differentiation^35^. There was a burn-in of 200 saved iterations. Among the 20 independent runs, a run with the highest log posterior probability was selected for further analysis. STRUCTURE 2.3.4. was run with 20 independent simulations for each value of K (1–10). The length of the burn-in was set to 100,000, and 100,000 MCMC iterations were run after the burn-in period. Admixture model and correlated frequency model were assumed. With the results, major modes on each K were obtained using CLUMPAK^69^. To determine the optimal number of K, Pritchard’s method using LnP(K)^67^ and Evanno’s ΔK method^70^ were both considered. STRUCTURE HARVESTER^71^ was used to obtain the results of the two methods. If clusters were detected, *F*_*ST*_ between the clusters was calculated using the analysis of molecular variance (AMOVA) in GenAlEx 6.503^61^, and statistical significance was determined using 999 permutations. Principal coordinate analysis (PCoA) was performed as an additional tool to validate the K value. PCoA was also conducted in GenAlEx 6.503^61^.

### Cone analysis and germination test

We selected three undamaged mature cones of each mother and measured their width and length. The cones were dried until the scales were naturally separated from the cone axis. The dried cones were weighed using a precision balance (QUINTIX 213-1SKR, Sartorius, Gottingen, Germany), then seeds were extracted and cleaned from the dried cones. After the cleaning, seed potential, percent developed seeds, percent damaged seeds, purity, and 100-seed weight were investigated^72–74^ (Supplementary Table S4).

All pure seeds were stored in a 3 ℃ refrigerator for 90 days during the winter for pre-germination chilling (pre-chilling). The seeds were placed on 90 mm Petri dishes with two layers of No.2 filter paper (Hyundai Micro, Anseong, Gyeonggi, Korea). After the pre-chilling, a germination test was conducted in March. Seeds were placed on two layers of No.2 filter paper in a Petri dish (Hyundai Micro, Anseong, Gyeonggi, Korea). The germination test was carried out for 28 days in a growth chamber (BF-600THG, BioFree, Seoul, Korea) in March 2022. The growth chamber was maintained at 20 °C temperature and 50% of humidity. Eleven hours of dark and 13 h of light were provided every day. Seeds were counted as germinated when a radicle or cotyledon was developed over 1 mm. After the test, germination percentage, mean germination time, and germination energy was calculated (Supplementary Table S2). The germination energy was based on day 6, which was the day when the most seeds germinated. Seeds that failed to germinate were cut to investigate the existence of the embryo viability^18,75,76^. Sixteen seedlings of each mother tree were transplanted to the soil and tested for survivorship in April. Thirty mother trees were used except mother tree M12 (Fig. 4), which lacked germinated seedlings. A total of 480 seedlings were transplanted into pots of 3 cm width and 5 cm depth. Soil was sterilized with an autoclave before use. The seedlings were stored together in the laboratory and greenhouse at 20–30 °C. The survivorship was counted at 2 and 6 months after the transplantation. Seedlings were considered dead when the whole leaves turned brown.

### Statistical analysis

Statistical analysis was implemented to find the relationship among genetic, spatial, and seed characteristics in R 4.2.0^68^. Skewed data sets were normalized by log, cube, square, or square root transformation. The normality and equal-variance of the data sets were checked before the correlation test. Pearson’s correlation test was conducted to identify the relationship.

The analysis was conducted on two levels: (1) on cone characteristics and (2) on mother tree characteristics. On both levels, we aimed to find which observable characteristics are related to the seed quality and quantity. We used purity and viability to quantify seed quality: $seed \;quality = normalized \;purity \times viability$, and considered this value as a comparative indicator. On mother trees, we aimed to find whether genetic or spatial factors also affect the seed quality. To quantify genetic relatedness, two methods were considered in this study: (1) genetic distance and (2) the number of neighboring trees. The genetic distance^62^ between individuals was calculated using GenAlEx 6.503^60^. With the result, the sum of genetic distance to all potential pollen donors was computed on each mother tree. If the value was higher than the others, we considered that tree was more related to overall individuals. The number of neighboring trees was counted based on the result of spatial autocorrelation analysis. Trees within the distance of genetic relatedness were counted on each mother tree.

### Supplementary Information

Supplementary Information.

## Data Availability

Raw genotype data is available in the supplementary file (Supplementary Data S1). The datasets used during the current study are available from the corresponding author on reasonable request.

## References

[1] White, T. L., Adams, W. H. & Neale, D. B. *Forest Genetics* 179–186, 272–277 (CABI, 2007).

[2] Epperson, B. K. Spatial patterns of genetic variation within plant populations. In *Population Genetics And Germplasm Resources in Crop Improvement* (eds. Brown, A. H. D. *et al*.) (Sinauer Associates, 1989)

[3] Pandey M, Gailing O, Hattemer HH, Finkeldey R (2011). Fine-scale spatial genetic structure of sycamore maple (*Acer pseudoplatanus* L.). Eur. J. For. Res..

[4] Yao X, Zhang J, Ye Q, Huang H (2011). Fine-scale spatial genetic structure and gene flow in a small, fragmented population of *Sinojackia rehderiana* (Styracaceae), an endangered tree species endemic to China. Plant Biol..

[5] Hardy OJ, Vekemans X (1999). Isolation by distance in a continuous population: Reconciliation between spatial autocorrelation analysis and population genetics model. Heredity.

[6] Frantz AC, Cellina S, Krier A, Schley L, Burke T (2009). Using spatial Bayesian methods to determine the genetic structure of a continuously distributed population: Clusters or isolation by distance?. J. Appl. Ecol..

[7] Blair C (2012). A simulation-based evaluation of methods for inferring linear barriers to gene flow. Mol. Ecol. Resour..

[8] François O, Durand E (2010). Spatially explicit Bayesian clustering models in population genetics. Mol. Ecol. Resour..

[9] Bagnoli, F. *et al*. Neutral patterns of genetic variation and applications to conservation in conifer species. In *Genetics, Genomics and Breeding Of Conifers* (ed. Plomion, C., Bousequet, J. & Kole, C.) (CRC press, 2011).

[10] Wagner AP, Creel S, Kalinowski ST (2006). Estimating relatedness and relationships using microsatellite loci with null alleles. Heredity.

[11] Glaubitz, J. C. & Moran, G. F. Genetic tools: The use of biochemical and molecular markers. In *Forest Conservation Genetics: Principles and Practice* (eds. Young, A. *et al*.) 42–53 (CSIRO, CABI, 2000).

[12] Garner BA, Hoban S, Luikart G (2020). IUCN Red List and the value of integrating genetics. Conserv. Genet..

[13] Hvilsom, C. *et al. Selecting Species and Populations for Monitoring of Genetic Diversity* (IUCN, 2022).

[14] IUCN/SSC. *Guidelines on the Use of Ex Situ Management for Species Conservation, ver. 2.0* (IUCN Species Survival Commission, 2014).

[15] Brown, A. H. D. & Marshall, D. R. A basic sampling strategy: Theory and practice. In *Collecting Plant Genetic Diversity: Technical Guidelines* (eds. Guarino, L. *et al*.) 75–91 (International Plant Genetic Resources Institute (IPGRI); CABI, 1995).

[16] Heybroek HM (1984). Clones in forestry and in nature. Arboric. J..

[17] Brown, A. H. D. & Hardner, C. M. Sampling the gene pools of forest trees for ex situ conservation. In *Forest Conservation Genetics: Principles and Practice* (eds. Young, A. *et al*.) (CSIRO, 2000).

[18] Godefroid S, Van de Vyver A, Vanderborght T (2009). Germination capacity and viability of threatened species collections in seed banks. Biodivers. Conserv..

[19] Williams CG (2009). Conifer Reproductive Biology.

[20] Farjon, A. *A Handbook of The World's Conifers (Volume 2): Revised and Updated Version* (Brill, 2017).

[21] Farjon, A. & Filer, D. *An Atlas of The World’s Conifers: An Analysis of Their Distribution, Biogeography, Diversity and Conservation Status* (Brill, 2013).

[22] Zhang, D., Katsuki, T. & Rushforth, K. *Abies nephrolepis*. The IUCN Red List of Threatened Species 2013: e.T42292A76095986. 10.2305/IUCN.UK.2013-1.RLTS.T42292A76095986.en (2013).

[23] Korea Forest Service. Save Korean endemic conifers, such as *Abies koreana*, from extinction crisis (2021, accessed 19 Oct 2021). https://www.korea.kr/news/pressReleaseView.do?newsId=156476006.

[24] Yun JH (2018). Vulnerability of subalpine fir species to climate change: Using species distribution modeling to assess the future efficiency of current protected areas in the Korean Peninsula. Ecol. Res..

[25] Woo LS, Hoon YB, Don HS, Ho SJ, Joo LJ (2008). Genetic variation in natural populations of *Abies nephrolepis* Max. in South Korea. Ann. For. Sci..

[26] Hong YP, Ahn JY, Kim YM, Yang BH, Song JH (2011). Genetic variation of nSSR markers in natural populations of *Abies koreana* and *Abies nephrolepis* in South Korea. J. Korean For. Soc..

[27] Seo HN, Park JH, Lim HI (2023). Selection of *Abies nephorlpis* materials for restoration for genetic diversity in Mt. Gariwangsan degraded area. Sustainability.

[28] Yang JC, Yi DK, Joo MJ, Choi K (2015). Phylogeographic study of *Abies koreana* and *Abies nephrolepis* in Korea based on mitochondrial DNA. Korean J. Pl. Taxon..

[29] Dakin EE, Avise JC (2004). Microsatellite null alleles in parentage analysis. Heredity.

[30] Song JH, Lee JJ, Kang KS (2008). Variation in cone, seed and bract morphology of *Abies nephrolepis* (Trautv.) Maxim and *A. koreana* Wilson in native forests. J. Korean For. Soc..

[31] Kim, J. W. & Yoon, J. K. *Forest Tree Seeds and Nursery Practice* 26 (Forestry Research Institute, 1994).

[32] Cullingham CI (2020). Confidently identifying the correct K value using the DeltaK method: When does K = 2?. Mol. Ecol..

[33] Guillot G, Leblois R, Coulon A, Frantz AC (2009). Statistical methods in spatial genetics. Mol. Ecol..

[34] Latch EK, Dharmarajan G, Glaubitz JC, Rhodes OE (2006). Relative performance of Bayesian clustering software for inferring population substructure and individual assignment at low levels of population differentiation. Conserv. Genet..

[35] Guillot G (2008). Inference of structure in subdivided populations at low levels of genetic differentiation-the correlated allele frequencies model revisited. Bioinformatics.

[36] Guillot G, Estoup A, Mortier F, Cosson JF (2005). A spatial statistical model for landscape genetics. Genetics.

[37] Tucker JM, Schwartz MK, Truex RL, Wisely SM, Allendorf FW (2014). Sampling affects the detection of genetic subdivision and conservation implications for fisher in the Sierra Nevada. Conserv. Genet..

[38] Basto MP (2016). Assessing genetic structure in common but ecologically distinct carnivores: The stone marten and red fox. PLoS ONE.

[39] Vekemans X, Hardy OJ (2004). New insights from fine-scale spatial genetic structure analyses in plant populations. Mol. Ecol..

[40] Lian C (2008). Nuclear and chloroplast microsatellite analysis of *Abies sachalinensis* regeneration on fallen logs in a subboreal forest in Hokkaido, Japan. Mol. Ecol..

[41] Paluch J, Zarek M, Kempf M (2019). The effect of population density on gene flow between adult trees and the seedling bank in *Abies alba* Mill.. Eur. J. For. Res..

[42] Major EI (2021). Fine-scale spatial genetic structure across the species range reflects recent colonization of high elevation habitats in silver fir (*Abies alba* Mill.). Mol. Ecol..

[43] Latouche-Halle C, Ramboer A, Bandou E, Caron H, Kremer A (2003). Nuclear and chloroplast genetic structure indicate fine-scale spatial dynamics in a neotropical tree population. Heredity.

[44] Jacquemyn H, Brys R, Vandepitte K, Honnay O, Roldan-Ruiz I (2006). Fine-scale genetic structure of life history stages in the food-deceptive orchid *Orchis purpurea*. Mol. Ecol..

[45] Jones FA, Hubbell SP (2006). Demographic spatial genetic structure of the Neotropical tree, *Jacaranda copaia*. Mol. Ecol..

[46] Piry S, Luikart G, Cornuet J-M (1999). Computer note. BOTTLENECK: A computer program for detecting recent reductions in the effective size using allele frequency data. J. Heredity.

[47] Korea Meteorological Administration. KMA Weather Data Service Open MET Data Portal (2022, accessed 23 Aug 2022) http://data.kma.go.kr.

[48] Singh RP, Prasad PV, Reddy KR (2013). Impacts of changing climate and climate variability on seed production and seed industry. Adv. Agron.

[49] Maity A, Pramanik P (2013). Climate change and seed quality: An alarming issue in crop husbandry. Curr. Sci..

[50] Owens JN, Takaso T, Runions CJ (1998). Pollination in conifers. Trends Plant Sci..

[51] Wong MH, Duan C, Long Y, Luo Y, Xie G (2013). How will the distribution and size of subalpine *Abies georgei* forest respond to climate change? A study in northwest Yunnan, China. Phys. Geogr..

[52] Tanaka N (2012). Predicting the impact of climate change on potential habitats of fir (*Abies*) species in Japan and on the East Asian continent. Procedia Environ. Sci..

[53] Su J (2018). Recent fragmentation may not alter genetic patterns in endangered long-lived species: Evidence from *Taxus cuspidata*. Front. Plant Sci..

[54] Liguo, F., Nan, L. & Mill, R.R. PINACEAE. In *Flora of China. Vol. 4 (Cycadaceae through Fagaceae)* (eds. Wu, Z. Y. & Raven P. H.) 11–52 (Science Press; Missouri Botanical Garden Press, 1999).

[55] QGIS Development Team. QGIS Geographic Information System, Open source geospatial foundation. http://qgis.org. (2009).

[56] National Geographic Information Institute. Contour line. National Spatial Data Infrastructure Portal-Open market (2022, accessed 13 Mar 2023). http://data.nsdi.go.kr/dataset/20180927ds0069.

[57] Hong JK, Lim J, Lee BY, Kwak M (2016). Isolation and characterization of novel microsatellites for *Abies koreana* and *A. nephrolepis* (Pinaceae). Genet. Mol. Res..

[58] Lian C, Goto S, Hogetsu T (2007). Microsatellite markers for Sachalin fir (*Abies sachalinensis* Masters). Mol. Ecol. Notes.

[59] Kearse M (2012). Geneious Basic: An integrated and extendable desktop software platform for the organization and analysis of sequence data. Bioinformatics.

[60] Van Oosterhout C, Hutchinson WF, Wills DPM, Shipley P (2004). MICRO-CHECKER: Software for identifying and correcting genotyping errors in microsatellite data. Mol. Ecol. Notes.

[61] Peakall R, Smouse PE (2012). GenAlEx 6.5: Genetic analysis in Excel. Population genetic software for teaching and research-an update. Bioinformatics.

[62] Van Rossum, G. & Drake, F. L. *Python 3 Reference Manual* (CreateSpace, 2009).

[63] Smouse PE, Peakall R (1999). Spatial autocorrelation analysis of individual multiallele and multilocus genetic structure. Heredity.

[64] Hardy OJ, Vekemans X (2002). SPAGeDi: A versatile computer program to analyse spatial genetic structure at the individual or population levels. Mol. Ecol. Notes.

[65] Loiselle BA, Sork VL, Nason J, Graham C (1995). Spatial genetic structure of a tropical understory shrub, *Psychotria officinalis* (Rubiaceae). Am. J. Bot..

[66] Guillot G, Mortier F, Estoup A (2005). Geneland: A computer package for landscape genetics. Mol. Ecol. Notes.

[67] Pritchard JK, Stephens M, Donnelly P (2000). Inference of population structure using multilocus genotype data. Genetics.

[68] R Core Team. R: A language and environment for statistical computing. R Foundation for Statistical Computing, Vienna, Austria. https://www.R-project.org/ (2022).

[69] Kopelman NM, Mayzel J, Jakobsson M, Rosenberg NA, Mayrose I (2015). Clumpak: A program for identifying clustering modes and packaging population structure inferences across K. Mol. Ecol. Resour..

[70] Evanno G, Regnaut S, Goudet J (2005). Detecting the number of clusters of individuals using the software STRUCTURE: A simulation study. Mol. Ecol..

[71] Earl DA, vonHoldt BM (2012). STRUCTURE HARVESTER: A website and program for visualizing STRUCTURE output and implementing the Evanno method. Conserv. Genet. Resour..

[72] Bramlett, D. L. *et al. Cone Analysis of Southern Pines—A Guidebook* (USDA Forest Service, 1977).

[73] Bonner, F. T. & Karrfalt, R. P. *The Woody Plant Seed Manual* (USDA Forest Service, 2008).

[74] Korea National Forest Seed and Variety Center. Forest Seed Test and Inspection Guidelines. NFSV regulation no. 26 (2019, accessed 29 Jan 2022). https://www.law.go.kr/LSW/admRulLsInfoP.do?admRulSeq=2100000175792.

[75] Demir I, Ermis S, Mavi K, Matthews S (2008). Mean germination time of pepper seed lots (*Capsicum annuum* L.) predicts size and uniformity of seedlings in germination tests and transplant modules. Seed Sci. Technol..

[76] Domin M (2008). Germination energy and capacity of maize seeds following low-temperature short storage. Sustainability.

