# Supplementary material for: Spatial genetic structure and seed quality of a southernmost Abies nephrolepis population
Source: Sci Rep. 2023 Oct 27;13:18419. doi: 10.1038/s41598-023-45635-w (PMC10611809; doi:10.1038/s41598-023-45635-w)
Supplement: Supplementary file 1 — Supplementary Information. [file 41598_2023_45635_MOESM1_ESM.pdf]

Table S1. Genetic diversity indices of the 31 mother tree of *A. nephrolepis* at Mt. Hambaeksan, South Korea.

		A	A_E	H_O	H_E	F
Mother trees	Mean	10.222	6.120	0.853	0.821	-0.042
	SE	1.115	0.739	0.027	0.017	0.034

A; number of alleles, A_E; number of effective alleles, H_O; observed heterozygosity, H_E; expected heterozygosity, F; fixation index; SE, standard error

Table S2. Formulae for the analysis of cone and seed characteristics

Indicator	Computation
SP	$2 \times Fertile\ scales$
P-DS	$\frac{Total\ developed\ seeds}{Seed\ potential} \times 100$
P-DM	$\frac{Damaged\ seeds}{Total\ developed\ seeds} \times 100$
Purity	$\frac{Weight\ of\ pure\ seeds\ (g)}{Weight\ of\ total\ developed\ seeds\ (g)} \times 100$
GP	$\frac{Germinated\ seeds}{Tested\ seeds} \times 100$
MGT	$\frac{\sum nD}{\sum n}$
GE	$\frac{Germinated\ seeds\ until\ Day\ 6}{Tested\ seeds} \times 100$
Viability	$\frac{Viable\ Seeds}{Tested\ Seeds} \times 100$

SP; seed potential, P-DS; percent developed seeds, P-DM; percent damaged seeds, GP; Germination percentage, MGT; mean germination time, D; days from the beginning of the germination test, n; number of seeds newly germinated at time D, GE; germination energy

Table S3. Age estimation from the cores extracted from two trees in different diameter class of the population of *A. nephrolepis*is at Mt. Hambaeksan, South Korea

Tree No.	DBH (cm)	Core No.	Height of core extraction (cm)	Calendar year of the first tree ring
M29	15.2	A	36	1971
		B	52	1974
M30	21.6	A	71	1985
		B	47	1976

Core of M29 were extracted near the pith, and cores of M30 were relatively farther from the pith. DBH; diameter at breast height

Table S4. Characteristics of nine microsatellite loci selected for the study

Locus	Primer sequences (5' to 3')	Repeat motif	Ta (°C)	GenBank Accession No.
AK87	F: GCAGCCTTATCTTCATTTTGTC<br>R: CACTTGAGCCACACTTGAACTA	$(TG)_N$	58	KP289899
AK171	F: GGCATTTGAACACTTACACTGA<br>R: AGATTTTTGTTGGAATCTGCAC	$(TG)_N$	58	KP869867
AK173	F: GAGACTAGCATATACACCATCGG<br>R: AAGGGAATACACTCAGTCGAGA	$(CA)_N$	58	KP289900
AK176	F: TTACACCGTTAAAAAGGGAATG<br>R: CTCATGATGTGTAGCCATTTGT	$(TG)_N$	58	KP869869
AK240	F: AGAGAAGGGTCGAGGAATTATC<br>R: GAAAGTAGCAAGTGTAACTTATGC	$(CA)_N$	58	KP869872
AK246	F: TAGATTGGCATATTGGACATCA<br>R: ATAGGTTGTTGAGCTGGATGTT	$(TG)_N$	58	KP869873
AK247	F: GGATGGTGCTTTGTTGATATTT<br>R: AAATGGTTTGAGCAACATTCTT	$(TG)_N$	58	KP869874
AK252	F: TGCATGTTGTTAGTTGGTAAGG<br>R: TCTAGGTGGAGCAACAAGAGAT	$(TG)_N$	58	KP869875
As13	F: ATGCAAGCAACCATCGATATG<br>R: GTTTCTTCCATAGAACACCTC	$(TG)_N$	55	AB290134
As20	F: TCTTGCAACGAGGGGATCCATAACCTG<br>R: CTAAGCATTGAGCCACATAATTC	$(TG)_N$	55	AB290136

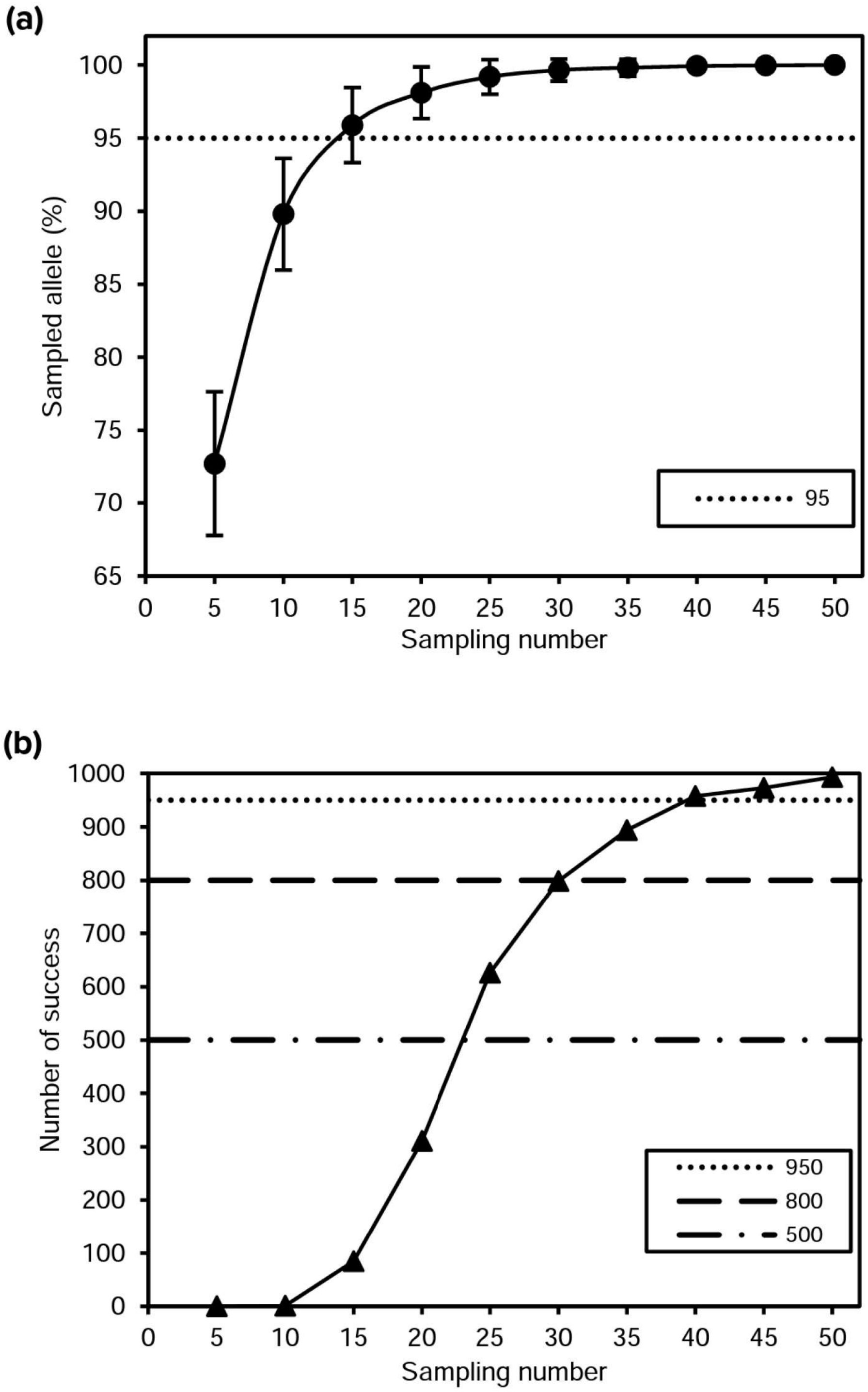

Figure S1. Results of the sampling simulation in the population of *A. nephrolepis* at Mt. Hambaeksan, South Korea. (a) 'Sampled allele (%)' indicates the percentage of sampled common alleles averaged over loci in 1,000 samplings, error bars indicate standard deviations, and (b) 'Number of success' represents the number of samplings that included over 95% of the common alleles at every locus.

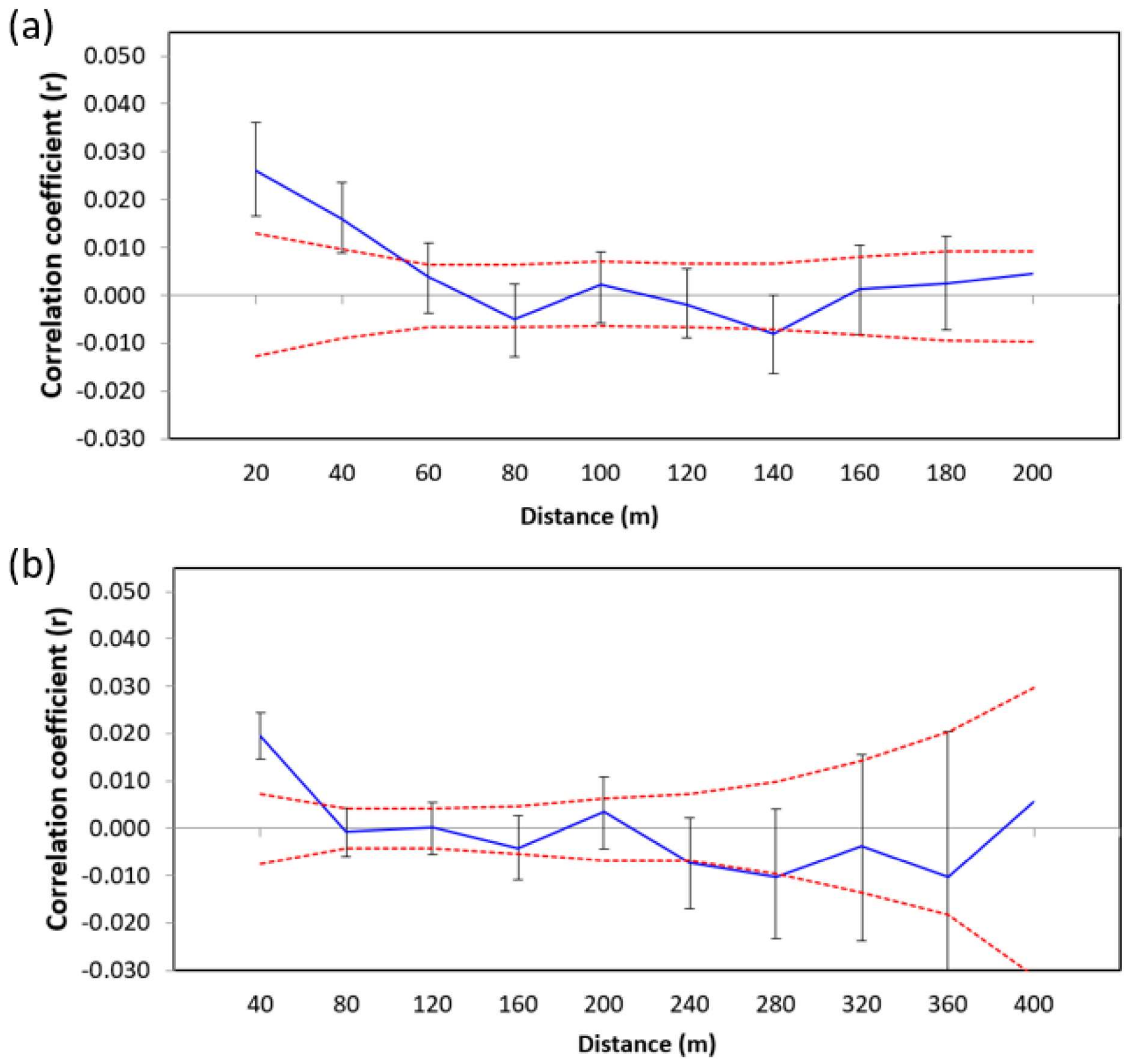

Figure S2. Correlograms of the population of *A. nephrolepis* at Mt. Hambaeksan, South Korea in 20 m and 40 m distance intervals . The blue line represents values of correlation coefficient (r) against the geographical distance between individuals with black error bars of 95% confidence interval. The red dotted line indicates the upper and lower limits of the null hypothesis with a 95% confidence interval. (a) Correlogram in 20 m distance interval, and (b) correlgram in 40 m distance interval.

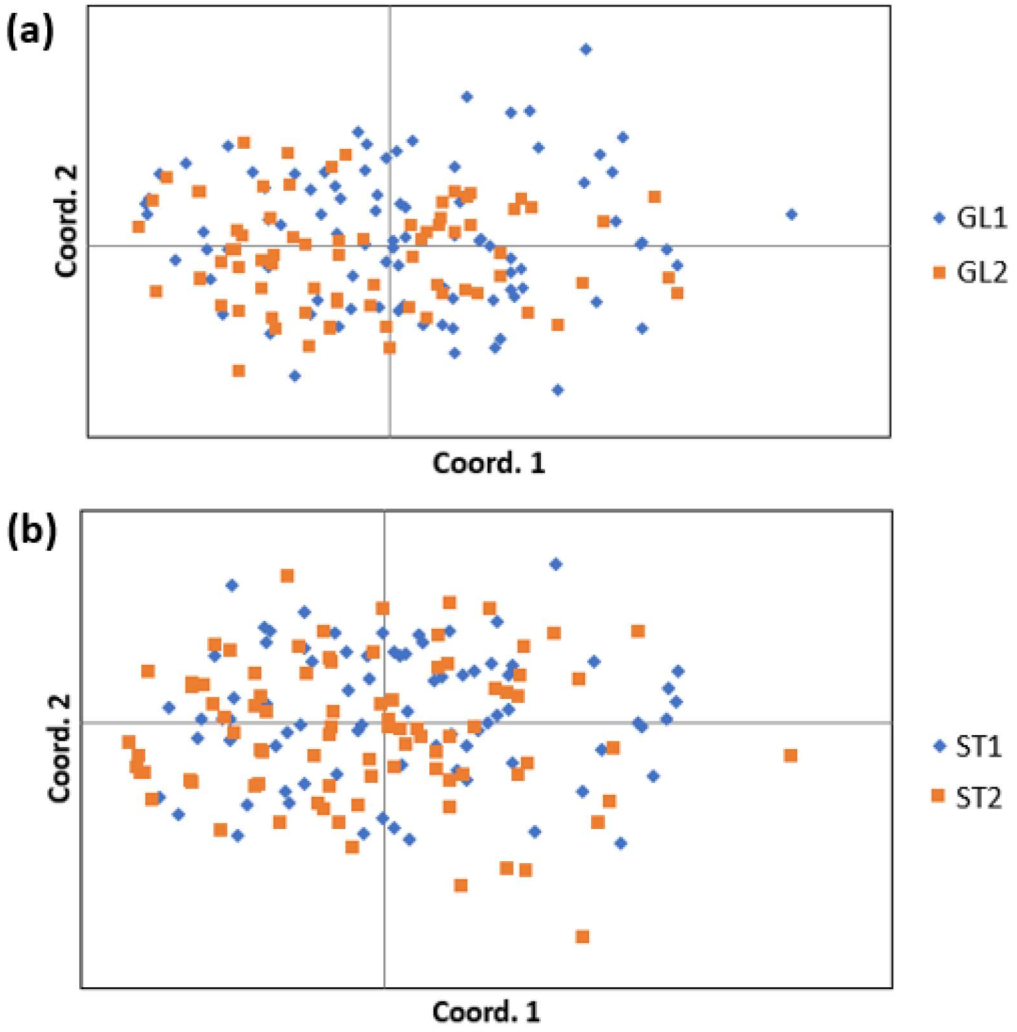

Figure S3. Results of principal coordinate analysis (PCoA) with the results of two Bayesian clustering models in the population of *A. nephrolepis* at Mt. Hambaeksan, South Korea. (a) a result of PCoA with two clusters estimated from GENELAND, and (b) a result of PCoA with two clusters estimated from STRUCTURE by Evanno's ΔK method.

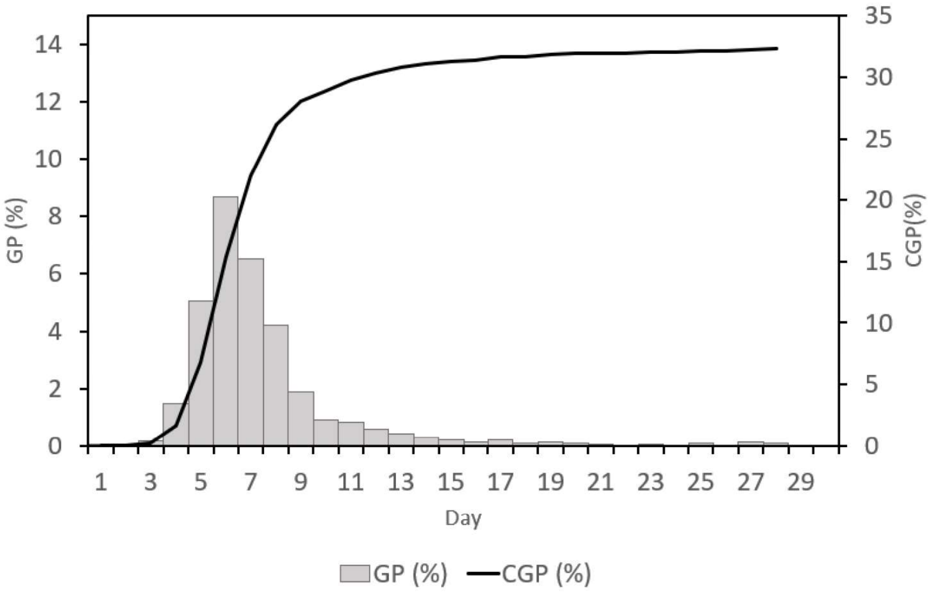

Figure S4. Germination curve of *A. nephrolepis* seeds from the population at Mt. Hambaeksan, South Korea. GP stands for germination percentage on each day, and GCP stands for cumulative germination percentage

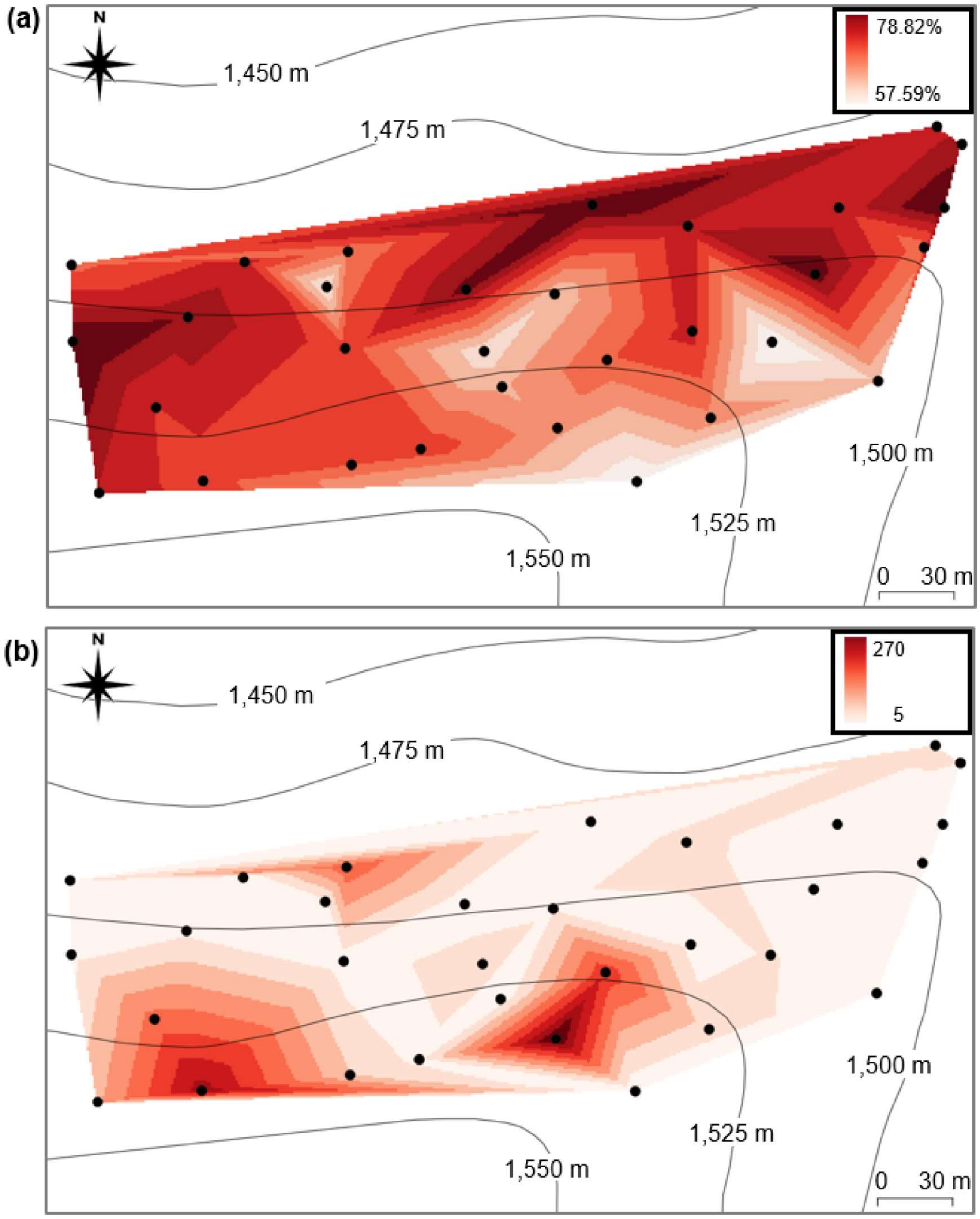

Figure S5. Interpolation of purity and cone abundance over the study plot at Mt. Hambaeksan, South Korea. Map was obtained by triangulated irregular network (TIN) interpolation in QGIS 3.28 [1]. (a) map of purity, and (b) map of cone abundance. Map of contour line was created by National Geographic Information Institute [2].

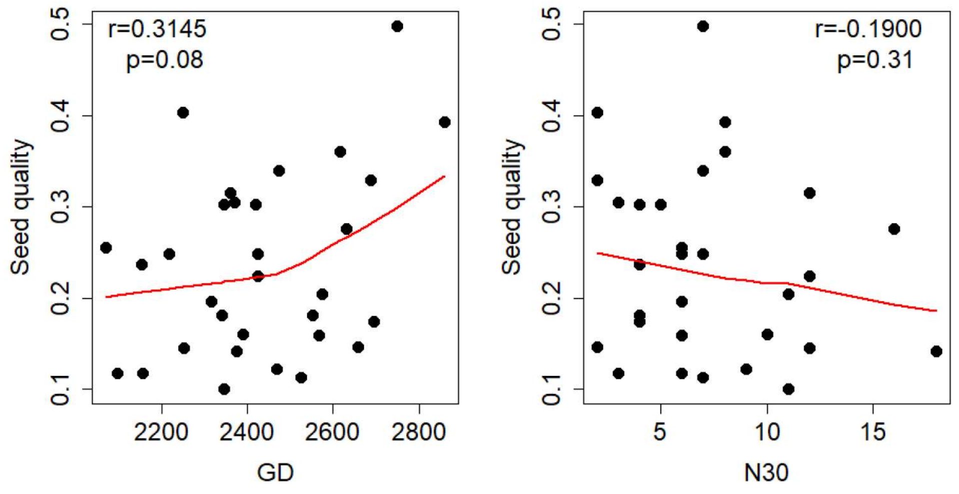

Figure S6. Scatterplots on seed quality and genetic relatedness of the mother trees in the population of *A. nephrolepis* at Mt. Hambaeksan, South Korea. GD represents the sum of genetic distance to potential pollen donors. N30 represents the square-rooted number of trees within a 30 m radius.

References

1. QGIS Development Team. QGIS Geographic Information System, Open source geospatial foundation. http://qgis.org. (2009).

2. National Geographic Information Institute. Contour line. National Spatial Data Infrastructure Portal-Open market. http://data.nsdi.go.kr/dataset/20180927ds0069. Accessed 13 March 2023. (2022).

Data S1. Raw data of genotypes and geographic coordinates (UTM52N) of sampled *A. nephrolepis* at Mt. Hambaeksan, South Korea.

Name	AK171	AK171	As13	As13	AK173	AK173	AK87	AK87	AK246	AK246	AK252	AK252	AK247	AK247	AK176	AK176	As20	As20	X	Y
M01	232	232	254	256	190	206	300	306	172	174	321	323	190	202	342	344	192	226	370691.9	408691.7
M02	232	232	252	256	204	204	294	306	168	174	319	319	196	200	338	338	192	212	370720.9	408723.3
M03	232	236	254	254	200	202	298	308	172	172	305	321	198	202	338	352	204	204	370787.6	408742.5
M04	232	234	256	258	184	208	308	322	170	172	323	323	194	198	350	352	192	200	370804.7	408807.7
M05	232	234	254	260	184	200	306	324	172	174	321	323	194	196	352	352	192	200	370812.4	408827.1
M06	234	236	254	256	184	198	280	300	166	170	319	321	194	202	342	344	192	204	370818.8	408857.9
M07	234	238	252	262	200	202	302	308	168	174	321	321	194	206	338	340	192	192	370808.6	408866.3
M08	230	246	252	256	204	206	296	308	172	174	321	321	204	204	352	354	192	224	370770.2	408826.2
M09	230	234	254	258	188	200	296	302	168	172	315	315	198	200	338	352	192	208	370761.3	408793.7
M10	230	232	256	256	184	202	298	306	172	172	313	321	196	202	344	352	192	204	370744.7	408760.6
M11	232	232	246	254	184	198	300	304	166	174	313	321	194	194	338	338	200	204	370678.8	408750.6
M12	232	232	252	256	184	184	292	300	166	170	309	317	190	198	338	352	192	214	370659.7	408717.2
M13	230	230	254	262	184	186	292	298	166	170	321	321	194	200	344	352	192	200	370636.9	408736.6
M14	232	236	252	254	186	200	302	322	172	174	313	321	194	196	342	352	192	200	370629.3	408753.9
M15	230	234	260	264	178	184	306	322	166	170	309	323	188	190	338	344	192	192	370621.5	408783.6
M16	236	242	250	262	184	202	302	306	168	174	309	309	186	188	344	352	200	210	370657.2	408782.1
M17	228	228	258	260	186	200	294	296	166	172	315	319	188	200	338	352	192	228	370709.9	408816.1
M18	232	232	248	254	198	198	296	302	172	172	305	325	190	200	338	344	192	236	370712.7	408765.2
M19	232	234	258	260	194	202	300	306	170	170	323	323	186	190	344	352	192	200	370671.4	408825.6
M20	232	234	258	260	184	202	298	306	166	172	319	323	196	202	342	342	224	236	370573.8	408801.0
M21	232	232	246	258	198	200	306	306	170	176	321	321	188	200	338	342	192	220	370565.6	408783.7
M22	232	234	256	258	184	204	302	302	166	166	321	321	192	192	338	342	198	214	370532.5	408795.0
M23	230	236	250	254	186	188	294	322	166	172	315	321	198	200	352	352	192	226	370463.2	408792.1
M24	232	232	256	256	184	200	280	298	174	174	321	321	198	198	338	344	224	232	370464.5	408755.2
M25	232	232	254	256	186	206	304	310	166	172	321	321	198	204	344	344	200	226	370510.3	408767.9
M26	232	232	252	254	184	184	286	306	172	174	319	321	190	202	338	342	192	192	370498.4	408723.8
M27	230	236	252	258	184	200	292	296	170	172	321	321	198	198	344	352	192	226	370573.6	408754.1
M28	232	232	254	254	178	178	296	308	172	174	321	321	196	204	338	342	192	222	370604.9	408705.7
M29	232	234	254	254	184	202	286	288	172	174	309	317	186	196	344	344	200	204	370577.3	408697.5
M30	232	232	252	258	198	208	302	304	166	170	321	321	188	194	342	342	192	198	370518.0	408688.5
M31	230	238	254	256	184	188	280	298	166	168	315	321	200	204	352	352	192	226	370476.4	408681.9

-----Continued on next page-----

P001	234	236	254	256	200	200	294	310	166	172	321	321	196	202	342	344	200	226	370656.3	408703.9
P002	232	232	256	258	184	188	302	306	172	174	311	323	188	200	338	342	192	192	370664.6	408705.4
P003	232	238	254	260	184	202	306	310	170	174	315	315	194	200	342	352	192	210	370692.9	408718.1
P004	232	232	254	256	184	184	294	310	172	174	321	321	188	200	352	352	192	192	370683.5	408700.0
P005	232	232	256	258	184	184	280	296	166	166	315	315	190	200	342	342	192	200	370701.8	408686.7
P006	232	232	254	258	202	208	296	306	172	172	321	321	190	202	342	352	222	226	370733.9	408729.3
P007	232	232	256	260	184	210	280	310	166	172	319	321	198	202	350	352	192	208	370735.6	408736.6
P008	228	238	244	256	184	202	306	310	172	174	319	321	188	200	338	342	192	208	370715.1	408725.6
P009	232	234	256	260	188	198	306	308	166	174	319	321	194	196	342	352	192	200	370707.1	408738.6
P010	236	236	248	258	184	184	294	296	172	174	305	317	188	190	342	352	204	208	370697.3	408739.7
P011	232	232	244	258	182	198	302	306	172	172	321	321	186	200	344	352	192	198	370694.1	408735.4
P012	232	232	256	260	184	184	294	316	172	172	321	323	188	192	342	344	198	208	370693.2	408744.5
P013	232	232	256	258	198	200	280	282	166	172	321	323	188	188	342	344	192	200	370688.2	408747.2
P014	232	232	252	258	190	190	300	322	170	172	319	319	198	200	338	342	192	204	370674.6	408731.5
P015	232	232	254	260	184	186	294	300	170	172	319	321	190	202	342	354	200	226	370659.6	408733.3
P016	230	236	246	258	176	198	294	300	172	172	319	321	198	200	338	344	200	226	370684.6	408753.1
P017	232	232	258	258	184	188	294	294	166	170	319	319	186	188	342	352	192	192	370675.7	408749.0
P018	232	238	258	258	184	204	280	304	166	172	319	321	186	200	338	342	192	236	370669.0	408745.8
P019	232	236	254	256	184	184	282	298	166	170	319	321	188	194	342	352	204	208	370668.1	408751.3
P020	220	234	256	258	184	184	286	298	172	172	321	321	194	194	338	352	192	200	370663.1	408749.1
P021	230	234	252	260	200	204	288	294	166	174	313	319	188	198	352	354	192	226	370651.3	408746.0
P022	230	232	256	260	186	198	298	302	166	174	309	321	194	194	338	350	192	222	370647.9	408742.4
P023	228	230	252	256	184	208	304	308	166	172	315	317	188	194	342	352	200	204	370633.8	408748.6
P024	236	236	252	256	176	198	294	302	172	172	315	321	194	198	344	352	200	230	370656.8	408753.7
P025	230	234	252	258	184	206	306	314	172	174	321	323	188	190	344	344	192	204	370676.3	408783.9
P026	230	236	262	264	182	184	296	312	170	172	309	317	194	196	342	352	214	224	370696.3	408792.5
P027	232	232	258	260	202	202	294	306	172	174	321	323	196	202	338	342	192	192	370693.9	408793.5
P028	232	232	252	264	184	200	304	306	166	174	315	321	190	190	342	352	192	200	370694.1	408761.3
P029	232	236	252	256	184	198	304	306	166	172	317	323	190	200	342	344	212	236	370696.8	408771.1
P030	232	234	256	262	186	202	296	322	168	170	309	321	196	196	342	344	214	226	370709.6	408769.1
P031	232	232	254	256	198	200	302	302	166	172	305	321	200	200	338	338	200	226	370708.8	408764.5
P032	232	232	248	254	186	198	294	302	166	172	309	309	188	188	344	352	192	198	370704.7	408746.4
P033	232	232	258	262	204	206	308	322	166	174	317	319	190	198	342	352	192	200	370732.7	408757.4
P034	232	244	256	258	202	204	286	306	166	174	323	325	186	200	338	352	192	222	370722.5	408764.6

-----Continued on next page-----

P035	232	242	254	260	182	182	296	308	166	174	319	321	190	196	342	352	208	228	370721.2	408770.7
P036	232	232	252	258	184	184	294	308	170	172	305	319	188	192	338	342	192	224	370757.6	408769.0
P037	224	232	252	262	200	202	308	312	166	168	309	317	186	186	342	352	192	208	370781.4	408754.7
P038	232	234	252	254	184	186	296	308	166	170	323	323	198	200	338	352	192	192	370786.5	408811.0
P039	232	232	252	258	202	208	294	306	166	174	321	323	186	188	352	352	192	204	370785.1	408737.5
P040	234	238	248	258	186	198	300	322	172	172	319	321	198	204	352	352	192	192	370801.8	408805.3
P041	232	232	254	256	198	208	302	308	172	172	305	305	190	190	338	352	192	192	370779.2	408822.4
P042	234	234	252	254	176	204	296	302	172	174	325	325	186	202	352	352	192	224	370803.7	408822.5
P043	232	232	256	258	202	212	294	300	170	172	321	321	188	190	342	344	192	210	370822.3	408818.0
P044	232	232	256	258	184	206	280	296	172	174	305	305	190	192	344	352	192	192	370822.1	408858.9
P045	232	232	252	254	200	200	280	298	170	170	315	317	186	200	342	344	192	226	370767.8	408804.5
P046	220	234	254	260	188	206	296	298	172	174	305	305	196	200	342	352	192	200	370746.2	408791.9
P047	232	234	258	262	184	206	286	300	174	174	323	323	184	188	342	344	200	214	370758.8	408787.1
P048	236	236	252	254	186	208	294	300	172	174	321	321	188	190	342	352	200	208	370739.6	408817.6
P049	234	236	256	260	200	202	320	322	166	172	305	305	188	188	338	344	202	222	370692.7	408819.8
P050	234	234	256	258	186	204	292	300	166	166	315	321	190	196	338	352	192	236	370659.1	408832.5
P051	232	232	258	260	184	202	280	308	166	168	319	323	190	200	352	352	200	212	370652.2	408827.9
P052	232	232	254	260	184	188	296	314	166	172	323	323	190	190	338	352	212	212	370645.6	408821.7
P053	230	236	254	254	202	204	294	296	166	174	321	321	194	200	338	342	212	218	370643.8	408801.9
P054	232	232	252	256	200	206	292	302	166	172	319	319	188	196	342	344	192	200	370639.9	408789.8
P055	232	252	254	260	184	202	286	298	172	174	315	325	198	206	350	352	200	208	370623.1	408789.2
P056	230	236	250	260	200	202	306	306	172	172	309	309	190	206	342	344	192	226	370625.4	408766.7
P057	230	232	254	256	184	186	302	306	166	166	313	315	194	194	352	352	204	226	370659.3	408720.5
P058	232	238	256	258	186	190	294	302	170	172	321	321	190	196	342	354	200	212	370654.1	408714.9
P059	240	240	256	260	182	184	300	306	172	174	323	323	190	190	342	342	192	192	370653.1	408712.3
P060	232	236	252	258	184	200	294	310	170	172	319	319	194	198	342	342	192	210	370772.7	408806.5
P061	232	234	252	254	182	184	280	304	172	176	321	325	196	196	342	350	200	208	370461.3	408678.7
P062	232	232	256	262	202	202	308	310	166	174	321	321	186	196	338	342	192	208	370474.0	408685.3
P063	232	232	248	252	184	186	296	308	166	168	321	321	196	196	344	352	200	208	370479.0	408695.1
P064	228	250	256	258	186	216	306	308	166	166	309	317	196	200	342	352	204	218	370481.4	408695.3
P065	232	232	246	252	204	216	296	304	166	170	309	309	190	202	342	344	192	192	370475.7	408705.1
P066	228	228	254	256	184	202	306	306	170	170	321	321	190	194	338	350	192	200	370464.5	408705.3
P067	232	236	256	258	182	184	280	306	166	172	313	313	198	198	352	354	192	222	370474.4	408732.8
P068	230	232	252	252	186	202	296	306	172	172	313	313	190	198	342	352	204	226	370463.0	408742.6

-----Continued on next page-----

P069	232	232	252	252	184	210	294	296	168	174	321	327	190	196	338	344	192	200	370489.2	408753.6
P070	232	232	252	262	184	204	298	302	166	168	321	321	186	198	344	344	200	208	370496.4	408748.8
P071	232	232	254	258	184	198	304	306	176	176	315	323	186	194	342	344	192	214	370502.4	408749.1
P072	238	250	252	256	182	190	294	302	172	172	323	323	186	204	342	342	200	222	370507.9	408747.3
P073	232	232	256	258	184	186	290	306	166	176	321	323	188	194	338	352	200	216	370510.4	408744.2
P074	232	232	254	258	192	202	300	306	172	174	315	321	186	196	342	342	192	212	370526.9	408736.0
P075	236	236	256	258	188	202	296	306	170	172	317	323	196	198	342	350	208	226	370534.2	408762.3
P076	232	232	256	264	202	202	300	306	166	170	321	321	186	190	338	342	192	200	370530.1	408763.5
P077	232	236	246	254	188	202	282	308	170	176	321	323	200	200	352	352	204	222	370535.9	408771.2
P078	232	234	254	256	200	202	290	294	172	174	309	315	190	190	342	352	200	222	370513.8	408773.5
P079	242	242	256	256	186	192	302	306	166	172	317	323	188	200	338	342	204	222	370519.3	408785.2
P080	234	250	250	258	184	198	306	308	166	172	321	321	186	194	338	344	192	226	370541.7	408788.7
P081	242	250	254	262	200	204	306	314	166	174	321	321	186	202	338	352	218	228	370542.0	408792.6
P082	236	236	252	252	184	202	306	314	172	174	317	321	186	190	344	352	192	210	370521.8	408797.8
P083	232	232	252	260	186	204	294	298	166	166	321	323	186	190	352	352	198	222	370507.5	408803.2
P084	236	236	254	260	184	206	280	296	172	174	309	321	192	192	338	352	192	192	370477.7	408796.9
P085	242	242	256	262	200	216	296	308	166	172	319	321	198	202	342	344	208	224	370453.0	408806.7
P086	236	236	252	256	188	202	298	302	168	170	319	321	192	196	342	352	192	228	370441.9	408795.7
P087	232	232	256	256	184	212	294	306	170	172	317	317	200	204	344	350	192	200	370469.3	408772.0
P088	232	244	252	256	184	188	294	294	170	174	319	319	196	200	344	352	192	224	370467.8	408770.2
P089	236	252	254	260	190	190	290	308	170	172	309	323	188	198	344	350	222	226	370468.4	408764.8
P090	232	232	252	256	184	204	306	308	166	172	313	321	200	200	342	344	208	222	370453.4	408752.7
P091	230	234	252	258	184	200	298	310	166	174	309	317	196	198	350	352	192	200	370550.4	408812.7
P092	236	250	254	256	184	186	292	308	174	174	317	321	196	200	338	352	200	208	370553.6	408818.3
P093	232	250	252	256	198	198	296	302	172	172	321	321	186	202	352	354	192	216	370566.1	408816.3
P094	234	250	252	256	184	184	296	306	166	172	315	321	196	200	344	352	192	192	370569.0	408805.9
P095	232	232	254	260	184	190	296	306	174	174	317	321	186	204	342	342	192	192	370574.2	408798.8
P096	232	232	252	258	184	208	300	306	170	170	319	321	186	188	342	342	192	222	370581.7	408798.2
P097	234	234	252	272	184	210	296	308	172	172	317	321	188	196	342	344	200	224	370586.7	408794.4
P098	236	236	254	260	184	188	298	306	172	172	315	323	188	188	344	344	200	226	370590.0	408795.3
P099	232	232	258	262	202	206	300	322	170	174	315	319	186	202	342	354	192	200	370596.8	408795.5
P100	232	232	254	254	188	188	302	308	172	172	319	323	190	194	344	350	200	222	370590.0	408788.8
P101	232	236	254	260	184	184	298	306	172	172	315	323	194	200	342	344	200	208	370589.6	408787.7

-----Continued on next page-----

P102	232	232	252	254	182	186	294	308	166	172	317	323	196	206	340	344	192	222	370599.4	408784.5
P103	232	232	254	262	202	204	286	294	166	172	323	323	188	194	338	342	200	204	370596.5	408777.0
P104	232	232	254	254	184	206	300	304	172	172	309	315	192	202	342	344	192	226	370595.6	408768.3
P105	232	232	250	260	184	202	294	322	172	174	309	317	192	204	342	350	208	220	370576.1	408760.8
P106	224	236	258	262	184	204	290	300	166	172	319	323	196	198	338	342	192	212	370570.9	408752.7
P107	232	232	254	256	182	202	302	306	168	174	321	325	186	200	350	352	200	210	370575.3	408753.4
P108	224	232	254	260	182	188	294	302	174	174	315	315	186	196	350	352	200	214	370575.5	408758.0
P109	232	232	258	260	200	204	296	300	172	174	323	323	192	200	342	352	192	232	370578.3	408754.4
P110	232	232	252	254	184	188	306	308	170	172	319	319	186	196	350	352	212	220	370582.6	408753.0
P111	232	232	250	254	188	198	296	308	166	172	321	321	194	204	342	344	200	216	370586.7	408747.2
P112	230	232	254	264	184	202	286	294	166	172	309	309	190	200	342	352	192	200	370589.7	408748.8
P113	234	236	256	256	184	184	294	294	172	174	317	317	186	196	342	350	192	192	370577.1	408747.1
P114	230	234	256	260	184	198	296	308	166	172	321	321	192	198	342	350	200	208	370573.1	408750.7
P115	232	232	254	260	184	200	308	308	172	174	319	321	192	192	340	350	214	224	370570.6	408744.2
P116	232	232	254	254	186	188	280	302	172	172	319	323	200	202	338	342	192	200	370577.1	408744.9
P117	232	232	254	264	188	188	280	308	166	170	317	321	196	200	338	352	192	208	370574.4	408743.4
P118	232	232	258	258	186	200	294	300	172	172	321	321	194	200	344	354	192	200	370572.9	408738.5
P119	220	220	252	254	186	204	306	324	166	172	309	323	198	200	342	344	192	222	370588.4	408741.9
P120	222	238	252	258	188	202	294	302	168	172	319	321	186	198	340	342	200	226	370581.4	408731.1
P121	236	236	256	256	184	184	308	308	170	172	321	321	192	196	342	354	208	234	370566.3	408729.0
P122	232	236	256	258	176	200	306	308	170	170	321	321	192	196	338	344	212	236	370556.8	408725.0
P123	236	236	252	256	184	202	294	296	172	172	321	321	190	196	338	352	200	212	370536.6	408700.6
P124	232	232	254	256	184	200	298	322	174	174	317	319	194	196	344	352	192	200	370534.5	408712.5
P125	232	232	252	256	182	200	286	306	166	166	315	321	188	200	342	342	222	226	370636.4	408717.9
P126	232	234	254	260	192	200	292	306	172	172	323	327	194	194	342	344	200	236	370612.2	408714.0
P127	234	234	256	258	190	200	310	322	172	172	305	309	196	202	342	342	210	226	370609.3	408717.6
P128	238	238	254	256	182	188	294	296	172	174	319	319	186	196	352	354	200	208	370597.4	408731.0
P129	232	234	252	260	184	202	298	300	172	172	309	317	186	190	338	344	200	200	370599.6	408715.4
P130	236	236	254	258	184	194	306	308	170	170	315	315	198	204	342	352	192	192	370597.3	408709.3
P131	232	232	252	252	188	202	296	298	172	172	309	317	192	200	344	354	192	200	370584.2	408699.0
P132	232	238	256	258	188	200	306	310	170	170	321	323	198	198	338	338	192	212	370577.0	408694.8
P133	230	232	252	254	198	206	288	300	166	174	313	315	194	198	342	352	192	192	370577.7	408704.0
P134	232	232	252	256	186	202	296	310	172	174	319	323	190	194	342	352	192	200	370496.3	408695.0
